# Supplementary material for: Globalization and social distance: Multilevel analysis of attitudes toward immigrants in the European Union
Source: PLoS One. 2022 Oct 3;17(10):e0274988. doi: 10.1371/journal.pone.0274988 (PMC9529102; doi:10.1371/journal.pone.0274988)
Supplement: S1 Table — (PDF) [file pone.0274988.s001.pdf]

Appendix Table: Country level variables for the 27 EU countries

Country	N	Social distance[a] (1-4)	Economic globalization[b] (0-100)	Social globalization[b] (0-100)	Unemployment rate[c]	Right-wing populism[d]
Austria	1,009	2.12	82.47	88.56	5.75	25.20
Belgium	1,001	1.86	88.49	87.08	7.80	3.70
Bulgaria	1,027	2.71	77.39	77.18	7.63	16.47
Croatia	1,035	2.16	74.59	82.89	13.50	2.40
Cyprus	501	1.99	83.12	86.17	12.97	7.60
Czechia	1,026	2.32	83.12	82.80	3.96	9.20
Denmark	997	1.60	84.12	89.75	6.03	21.10
Estonia	1,000	2.16	86.12	85.37	6.24	8.30
Finland	1,015	1.92	82.40	86.19	8.94	17.90
France	1,023	1.70	77.63	86.33	9.94	14.00
Germany	1,551	1.94	79.68	87.40	4.16	8.20
Greece	997	2.20	71.94	81.73	23.31	11.90
Hungary	1,038	2.81	82.51	80.72	5.36	65.20
Ireland	1,006	1.42	88.38	89.26	8.33	0.00
Italy	1,024	2.25	69.32	81.15	11.60	5.50
Latvia	1,001	1.99	81.66	80.41	9.41	16.60
Lithuania	1,008	2.04	78.47	84.94	8.02	12.13
Luxembourg	507	1.58	88.57	92.03	6.16	6.60
Netherlands	1,013	1.30	89.33	86.74	5.91	13.77
Poland	1,035	2.22	73.02	79.73	6.18	46.40
Portugal	1,089	1.56	79.47	80.18	10.79	0.50
Romania	1,054	2.25	70.73	76.98	5.88	2.97
Slovakia	1,080	2.34	81.92	82.45	9.76	17.00
Slovenia	1,012	1.74	76.68	83.19	7.84	23.07
Spain	990	1.51	75.72	83.39	19.64	0.20
Sweden	1,042	1.32	83.20	90.06	7.05	12.90
UK	1,381	1.45	80.74	90.38	4.81	9.70
Total/Mean	27,462	1.94	80.16	84.49	8.59	14.13

Data sources:

[a] Eurobarometer (GESIS, Cologne. ZA6927 Data file Version 2.0.0, https://doi.org/10.4232/1.13918)

[b] KOF Globalisation Index (https://kof.ethz.ch/en/forecasts-and-indicators/indicators/kof-globalisation-index.htm)

[c] World Bank (https://databank.worldbank.org/home.aspx)

[d] Timbro Authoritarian Populism Index (https://populismindex.com/data/)
